# EHMTI-0024. The typical duration of migraine aura: a prospective diary-based study

**DOI:** 10.1186/1129-2377-15-S1-D71

**Published:** 2014-09-18

**Authors:** M Viana, M Linde, G Sances, N Ghiotto, E Guaschino, M Allena, G Nappi, PJ Goadsby, C Tassorelli

**Affiliations:** 1 Headache Science Center, C. Mondino National Neurological Institute, Pavia, Italy; 2 Norwegian National Headache Centre Department of Neuroscience, Norwegian University of Science and Technology, Trondheim, Norway; 3 Headache Group – NIHR-Wellcome Trust Clinical Research Facility, King's College London, Pavia, Italy

## Introduction

In ICHD-IIIβ, non-hemiplegic migraine aura (NHMA) duration is considered normal when each symptom is no longer than one hour. A recent systematic review of the topic (Viana et al., 2013) did not find any article exclusively focusing on the duration of the aura. The pooled analysis of data from the literature on aura duration showed that visual symptoms lasting for more than one hour occurred in 6%–10% of patients, sensory symptoms in 14%–27% and dysphasic symptoms in 17%–60%.

## Aim

To evaluate the duration and variability of individual symptoms of NHMA in a prospective diary-aided study.

## Methods

We recruited 136 consecutive patients affected by NHMA at the Headache Centers of Pavia and Trondheim. The study received the approval by the local Ethics Committees. All patients signed an informed consent form. All the patients prospectively recorded the characteristics of three consecutive attacks in an ad hoc aura diary that included the time of onset and the end of each aura symptoms and the headache.

## Results

Of the 136 patients recruited so far, 44 completed the diaries during three consecutive auras for a cumulative number of 132 auras recorded. Of the remaining 92 patients, 21 dropped-out and 71 have not completed three aura attacks. Visual symptoms lasted for more than one hour in 21 out of 129 auras (16%), somatosensory symptoms in 9 out of 47 auras (19%), dysphasic symptoms in 3 out of 15 auras (20%). Six patients out of 44 experienced the same aura symptoms lasting for more than one hour in one attack and for less than one hour in another attack out the three.

## Conclusions

Our preliminary data suggest the duration of single symptoms of NHMA may be longer than one hour in a significant proportion of migraineurs, and that the one-hour limit needs review.

**Figure 1:**
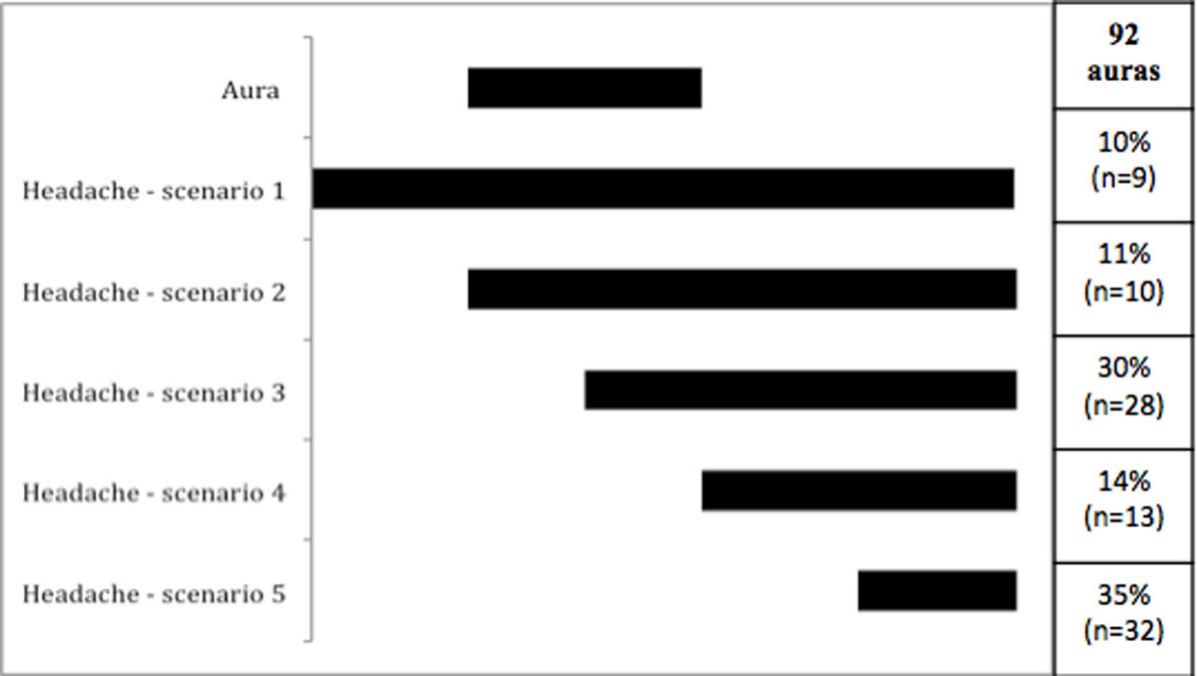


No conflict of interest.

